# *Cornus mas* L. Increases Glucose Uptake and the Expression of *PPARG* in Insulin-Resistant Adipocytes

**DOI:** 10.3390/nu14112307

**Published:** 2022-05-31

**Authors:** Małgorzata Małodobra-Mazur, Aneta Cierzniak, Martyna Ryba, Tomasz Sozański, Narcyz Piórecki, Alicja Z. Kucharska

**Affiliations:** 1 Department of Forensic Medicine, Division of Molecular Techniques, Wroclaw Medical University, Sklodowskiej-Curie 52, 50-369 Wrocław, Poland; aneta.cierzniak@umw.edu.pl (A.C.); martyna.ryba@wp.pl (M.R.); 2 Department of Pharmacology, Wroclaw Medical University, Jana Mikulicza-Radeckiego 2, 50-345 Wrocław, Poland; tomasz.sozanski@umw.edu.pl; 3 Institute of Physical Culture Sciences, Medical College, University of Rzeszów, Towarnickiego 3, 35-959 Rzeszów, Poland; npiorecki@ur.edu.pl; 4 Bolestraszyce Arboretum and Institute of Physiography, Bolestraszyce 130, 37-722 Wyszatyce, Poland; 5 Department of Fruit, Vegetable and Plant Nutraceutical Technology, Wroclaw University of Environmental and Life Sciences, J. Chełmońskiego 37, 51-630 Wrocław, Poland; alicja.kucharska@upwr.edu.pl

**Keywords:** *Cornus mas* L., insulin resistance, glucose utilization, adipocytes, *PPARG*

## Abstract

*Cornus mas* L., also known as cornelian cherry (CM), is a species that has long been cultivated in many different countries. In numerous scientific reports, cornelian cherry is used to treat numerous diseases and conditions. The presented study evaluated the effect of red and yellow *Cornus mas* L. extract on insulin sensitivity in adipocytes. 3T3-L1 fibroblasts as well as human SAT-derived and VAT-derived adipocytes were differentiated in vitro, and insulin resistance was induced using palmitic acid (16:0). The effect of CM fruit extract was analyzed in terms of glucose uptake and insulin signaling gene expression. In the glucose uptake test after insulin stimulation, a significant increase in glucose uptake was demonstrated in cells treated with CM fruit extracts. Furthermore, CM fruit extracts increased the expression of insulin signaling genes in adipocytes stimulated with insulin in control cells and adipocytes treated with CM extract. Additionally, a significant increase in peroxisome proliferator activated receptor gamma (*PPARG)* expression was observed in cells supplemented with CM extract. In conclusion, studies have shown that CM fruits can overcome insulin resistance and thus they have a positive effect on cell metabolism.

## 1. Introduction

According to the World Health Organization (WHO), diabetes is among the top ten causes of death in the world. Since 2000, the number of diabetes-related deaths has increased by 70%. It is estimated that 34.2 million people suffer from diabetes, of which 7.3 million remain undiagnosed [1]. Insulin resistance (IR) is a condition that involves lower sensitivity to insulin in peripheral tissues, which directly leads to type 2 diabetes pathogenesis [2]. The main causes of insulin resistance are obesity, lack of physical activity, and poor diet [3].

In the early stages of insulin resistance pathogenesis, patients are often asymptotic (without polyuria, polydipsia, or unintentional weight loss). Insulin resistance in most cases is diagnosed when compensatory mechanisms decline (i.e., insulin secretion from pancreatic β cells increases), at the stage of pre-diabetes or when full-blown type 2 diabetes is diagnosed. Treatment of patients who developed type 2 diabetes mellitus at the very early stages of the disease generally includes diet and lifestyle education to achieve normoglycemia via weight reduction, diet, and exercise. Further treatment requires evaluation for vascular or cardiovascular complications and the minimization of other long-term risk factors [4]. Initial pharmacological treatment generally begins with metformin [5] or other groups of drugs, including thiazolidinediones (rosiglitazone and pioglitazone), which act by abolishing insulin resistance while favorably affecting lipid metabolism.

*Cornus mas* L. (CM), also known as cornelian cherry (CC), belongs to the Cornaceae family that consists of 65 species, differing in appearance, ripening time, the basic chemical composition of the fruit, as well as the content of biologically active compounds and antioxidant activity [6,7]. CM has long been cultivated in many different countries, mostly in southern and central Europe, southwest Asia, eastern and western North America, and the mountains of Central America, South America, and East Africa [8]. Numerous studies confirm that CM is a rich source of biologically active compounds such as vitamin C, organic acids, pectic compounds, phenolic acids, flavonoids (anthocyanins, flavonols), and iridoids (loganic acid, cornuside). CM and its products are also a rich source of minerals such as potassium, calcium, phosphorus, sodium, magnesium, as well as iron, zinc, copper, and manganese. However, various parts of *C. mas* L. contain different amounts and different compositions of individual biologically active substances. Iridoids are found mainly in the leaves and young shoots of botanical plants, and very rarely in fruits, while flavonols and flavonoids, including anthocyanins, are found in many parts of the plants [6,9].

In numerous scientific reports, *Cornus mas* L. was used as the treatment agent for various diseases and conditions, including influenza, asthma, diarrhea, as well as gastrointestinal and metabolic disorders. It is also used as a wound-healing agent, anti-inflammatory agent, and appetizer [8]. It is also described as an important antimicrobial agent which showed significant activity against numerous bacteria and fungi including *Bacillus*, *E.coli*, *Staphyloccocus aureus*, and *Pseudomonas aeruginosa* [10,11]. The previous studies conducted by the authors showed lipid-lowering and antiatherosclerotic properties of CM fruits and their isolated constituents [12,13,14] as well as their inhibitory effect on endogenous nitric oxide antagonists [15,16]. *Cornus mas* L. has also been reported as an antiobesity and antidiabetic drug that improves metabolic homeostasis and overall carbohydrate metabolism [17,18]. There is a diverse range of CM usage as a treatment agent based on geographical distribution.

Most of the reported extracts obtained from CM were prepared based on fruits. Thus, the presented study evaluated the effect of red and yellow *Cornus mas* L. fruit extract on insulin sensitivity in adipocytes. An attempt was made to evaluate if CM fruit extract could reverse previously induced insulin resistance, thus standing as a potential therapeutic agent against insulin resistance and type 2 diabetes. Second, by using two various specimens with different content of biologically active compounds, e.g., anthocyanins, we wanted to see if there were differences in the health benefits of different types of CM fruit extracts. Lastly, the authors were trying to assess potential mechanisms through which CM fruit extract could restore proper insulin sensitivity.

## 2. Materials and Methods

The study protocol was approved by the Ethics Committee Board of Wroclaw Medical University, Approval No. KB-124/2017.

### 2.1. Reagents and Standards

All reagents and organic solvents were of analytical grade. Authentic standards of loganic acid, cyanidin 3-*O*-glucoside, *p*-coumaric acid, gallic acid, quercetin 3-*O*-glucoside, and kaempferol 3-*O*-glucoside were purchased from Extrasynthese (Genay Cedex, France). Trans-caftaric acid was purchased from the Cayman Chemical Company (Michigan, EUA, Ann Arbor, MI, USA). *Trans*-Coutaric acid was purchased from Merck (Darmstadt, Germany). Methanol, acetonitrile, and formic acid were obtained from POCh (Gliwice, Poland).

### 2.2. Conrus mas L. Fruits Extract Preparation

Yellow (mixture of two cultivars ‘Yantarnyi’ and ‘Flava’) and red (‘Podolski’ cv.) cornelian cherry fruits (*Cornus mas* L.) were harvested from the Arboretum in Bolestraszyce, near Przemyśl, Poland. The plant materials were authenticated by Elżbieta Żygała, M.Sc. (Arboretum and Institute of Physiography in Bolestraszyce, Przemyśl, Poland), and the adequate voucher specimens (‘Yantarnyi’—BDPA 14131; ‘Flava’—BDPA 8795; ‘Podolski’—BDPA 10462) were deposited at the Herbariums of the Arboretum in Bolestraszyce, Poland. After harvesting, fully ripe fruits were immediately frozen at −20 °C until further analysis. Next, frozen fruits were shredded and heated for 5 min at 95 °C using a Thermomix (Vorwerk, Wuppertal, Germany). The pulp was subsequently cooled down to 50 °C and depectinized at 50 °C for 2 h by adding 0.5 mL of Pectinex BE XXL (Novozymes A/S, Bagsværd, Denmark) per 1 kg. Following depectinization, the pulp was pressed in a laboratory hydraulic press (SRSE, Warsaw, Poland). The pressed juice was run through an Amberlite XAD-16 resin column (Rohm and Haas, Chauny Cedex, France). Impurities were washed off with distilled water. Extracts from red juice (Red Extract) and yellow juice (Yellow Extract) were eluted with 80% ethanol. The extracts were concentrated under a vacuum at 40 °C. The solvent was evaporated using the Rotavapor (Unipan, Warsaw, Poland) and then the extracts were freeze-dried (Alpha 1–4 LSC, Christ, Osterode am Harz, Germany).

### 2.3. Quantitative Determination of Compounds with the Use of HPLC-PDA

HPLC analysis was performed using a Dionex (Germering, Germany) system equipped with an Ultimate 3000 diode array detector, LPG-3400A quaternary pump, EWPS-3000SI autosampler, and TCC-3000SD thermostated column compartment, and controlled by the Chromeleon v.7.2 software (Thermo Scientific Dionex, Sunnyvale, CA, USA). The content of individual iridoids, anthocyanins, flavonols, phenolic acids, and isomers of hydrolyzable tannins was determined using the optimized HPLC-PDA methods (I and II) and then the sums of particular phytochemical groups were calculated. Compounds were identified through the comparison of their retention times and UV–Vis spectra to those of authentic standards and by relating them to the literature data. The extracted sample was analyzed in three repetitions. The results (sums of phytochemical groups) were expressed as mg/g dry weight (dw).

The content of iridoids, anthocyanins, flavonols, and phenolic acids was determined using method I, as described previously by Spychaj et al. [7]. The separation of compounds was achieved using the Cadenza CD-C18 column (75 × 4.6 mm, 5 μm) with a guard column (Imtakt, Kyoto, Japan). The mobile phase was composed of solvent A (4.5% aq. formic acid, *v*/*v*) and solvent B (100% acetonitrile). The gradient profile was as follows: 0–1 min 5% B in A, 1–20 min 25% B in A, 20–26 min 100% B, 26–30 min 5% B in A. The flow rate of the mobile phase was 1 mL/min, and the injection volume was 20 μL. The column was operated at 30 °C. Iridoids were detected at 245 nm, anthocyanins at 520 nm, flavonols at 360 nm, and phenolic acids at 320 nm. Calibration curves at concentrations in the range of 0.02–0.3 mg/mL (*R*^2^ ≥ 0.9998) were determined experimentally for loganic acid, cyanidin 3-*O*-glucoside, quercetin 3-*O*-glucoside, kaempferol 3-*O*-glucoside, caffeic acid, and *p*-coumaric acid. The quantification of hydrolyzable tannins was performed according to the method by Przybylska et al. [19]. The separation was carried out using the Hypersil GOLD C18-column (250 × 4.6 mm, 5 μm) with a guard column (Thermo Fisher Scientific Inc., Salt Lake City, IL, USA). The following mixtures were used as eluents: C, water-FA (98.5:1.5, *v*/*v*) and D, acetonitrile-FA (98.5:1.5, *v*/*v*). The gradient profile applied was as follows: initial conditions 100% C, 30 min; 30% D, 33 min; 70% D, 45 min; 70% D in C, 48 min; 100% D, 55–60 min; 100% C. The flow rate of the mobile phase was 1.2 mL/min, and the injection volume was 20 μL. The column was operated at 22 °C. Hydrolyzable tannins were detected at 280 nm. The calibration curve at concentrations in the range of 0.02–0.3 mg/mL (*R*^2^ ≥ 0.9996) was determined experimentally for gallic acid.

### 2.4. Cell line Culturing and Adipocyte Differentiation 

3T3-L1 fibroblasts (ATCC, CL-173™) were cultured in DMEM (Corning, Tewksbury, MA, USA) enriched with 10% FBS (Corning) and antibiotics mix including penicillin, 50 U/mL; streptomycin, 50 µg/mL, Corning. The medium was changed every second day until confluence. On the day of confluence, the differentiated medium, containing 10% fetal bovine serum (FBS), antibiotics (penicillin, 50 U/mL; streptomycin, 50 µg/mL), dexamethasone (390 ng/mL), 3-isobutyl-1-methylxanthine (115 µg/mL), and insulin (10 µg/mL, all purchased from Sigma-Aldrich, St. Louis, MO, USA) was added for three days, followed by medium sustaining differentiation containing DMEM with antibiotics, 10% FBS, and insulin (10 µg/mL) for additional three days. Next, the medium was changed to DMEM containing 10% FBS and antibiotics, and cells were further cultured for an additional two days. 3T3-L1 fibroblasts were differentiated into mature adipocytes after eight days from the initiation of differentiation. All experiments were performed in triplicates. 

Human mesenchymal stem cells (MSC) were extracted from the stromal cell fraction of adipose tissue biopsies collected from three healthy men at the age of 44 ± 5 years with normal BMI (23 ± 1.4 kg/m^2^), insulin (8.0 ± 1.4 µU/mL), glucose (94 ± 4 mg/dL), and lipid levels (TG 76 ± 11 mg/dL; HDL 60 ± 17 mg/dL; LDL 116 ± 29 mg/dL). From each enrolled patient, a pair of adipose biopsies were taken from subcutaneous adipose tissue (SAT) and visceral adipose tissue (VAT). 

The MSCs were extracted as described before [20]. Briefly, adipose tissue biopsies were collected on phosphate-buffered saline with a protease inhibitor mix for transport purposes, and then the tissue was dissected with scissors, blood vessels were removed and the tissue fragments were digested with collagenase (1 mg/mL medium, Sigma Aldrich) enriched with bovine serum albumin (10 mg/mL medium, Sigma-Aldrich) until complete digestion. Following several washing steps, MSCs were plated on the dishes in DMEM/F12 (50/50, Corning) medium supplemented with 10% FCS and antibiotics (penicillin, 50 U/mL; streptomycin, 50 µg/mL, Corning).

The medium was replaced every second day until confluence. At the confluence stage, differentiation cocktail containing DMEM/F12 (50:50, Corning), 10% FCS (Corning), penicillin (50 U/mL), streptomycin (50 µg/mL), IBMX (115 µg/mL), dexamethasone (390 ng/mL), insulin (10 µg/mL), pioglitazone (0.1 µg/mL) and human transferrin (10 µg/mL) (all purchased from Sigma-Aldrich) was added for three days. Next, the medium was replaced with a medium containing DMEM/F12 (50:50), 10% FCS, antibiotics, insulin (10 µg/mL), pioglitazone (0.1 µg/mL) and human transferrin (10 µg/mL), and it was cultured for the next three days. The adipogenesis was continued for additional three to four days using a medium containing DMEM/F12 (50:50) and 10% FCS and antibiotics. After the following three to four days, the cells were fully mature adipocytes. The differentiation was performed in duplicate for each of the extracted MCSs.

The adipogenesis of 3T3-L1 fibroblasts and human MSCs was controlled based on morphological changes observed under a microscope during differentiation (Supplementary Material, Figure S1) and by measuring the expression of transcription factor genes controlling adipogenesis, namely *PPARG* (Peroxisome Proliferator Activated Receptor Gamma) and *CEBPA* (CCAAT Enhancer Binding Protein Alpha, Supplementary Material, Figure S2).

The adipogenesis of 3T3-L1 fibroblasts and human MSCs was controlled based on morphological changes observed under a microscope during differentiation (Supplementary Material, Figure S1) and by measuring the expression of transcription factor genes controlling adipogenesis, namely *PPARG* (Peroxisome Proliferator Activated Receptor Gamma) and *CEBPA* (CCAAT Enhancer Binding Protein Alpha, Figure S2).

### 2.5. Viability Test (MTT)

The cells were seeded at a concentration of 3000 cells per well. Next, the *Cornus mas* L. fruit extract was added at concentrations ranging from 1 mg/mL up to 0.002 mg/mL. It was followed by a viability test performed after 24 h and 48 h of incubation using MTT (3-(4,5-Dimethylthiazol-2-yl)-2,5-diphenyltetrazolium Bromide, Sigma-Aldrich). DMSO (Sigma-Aldrich) was used as a control (solvent for extract resuspension). A 5 mg/mL MTT solution was added to the previously seeded cells with CM fruit extract and incubated for 3.5 h. Subsequently, the medium was removed and the metabolites of MTT were resuspended in DMSO, incubated for 15 min with gentle agitation, and measured at 590 nm with a reference filter of 620 nm. The number of viable cells was estimated based on the amount of formazan obtained in the process of oxidative phosphorylation, hence the survival of cells was indirectly dependent on the mitochondrial metabolism. The viability was assessed based on three independent experiments.

### 2.6. Insulin Resistance Induction and Glucose Uptake Measurements

Insulin resistance was induced using 0.5 mM palmitic acid (16:0, Sigma-Aldrich) for 48 h. Next, after IR induction, the appropriate concentration of CM fruit extract was added to the cells for an additional 48 h. Control cells were treated with DMSO. The day before the experiment, cells were starved in an FBS/FCS-free medium. On the day of the experiments, part of the cells was stimulated with 1 μM insulin (Sigma-Aldrich) for 15 min. Next, all cells were incubated with 1 mM of 2-deoxyglucose (as part of Glucose Uptake-Glo™ Assay, Promega Corporation) for an additional 15 min at RT. Then, the cells were further processed using a Glucose Uptake-Glo™ Assay according to the manufacturer’s protocol. The luminescence was measured using the Victor3, 1420 Multilabel Plate Reader (Perkin Elmer, Waltham, MA, USA). Glucose uptake in experimental and control cells was assessed based on three independent experiments.

### 2.7. RNA Extraction and Gene Expression Analysis

Total RNA was extracted from adipocytes using TriReagent (Sigma-Aldrich). Briefly, cells were harvested, centrifuged, and 1 mL of TriReagent was added to the cell pellet. Cells were incubated for 8 min on ice; next, 200 μL of chloroform (Sigma-Aldrich) was added, and samples were mixed vigorously and centrifuged for 20 min at 4 °C. The aqueous phase was transferred to a new Eppendorf tube and RNA was precipitated with isopropanol (Sigma-Aldrich) and centrifuged at the maximum speed for 15 min at 4 °C. The RNA pellet was washed with 70% of ethanol (Chempur, Karlsruhe, Germany), dried, and resolved in molecular biology-grade water. cDNA was obtained using the High Capacity Reverse Transcription Kit (ThermoFisher, Waltham, MA, USA). Gene expression was analyzed in Real-Time PCR with the use of Fast SYBR Green Master Mix (ThermoFisher). Primers were designed manually and the efficiency of primers was checked using the standard curve. For all sets of primers, the efficiency was close to R = 99%. The specificity of Real-Time PCR was checked with the use of melting curve analysis. The sequences and efficiency data of primers were published previously [21,22]. Normalization was carried out to the housekeeping gene (*β-actin*) and calculated according to the ΔΔCt algorithm. 

### 2.8. Statistical Analysis

Statistical analysis was conducted using Statistica13.1 (StatSoft, Tulsa, OK, USA). To analyze differences between groups, a *t*-test was used. The normality of the distribution of variables was checked using the Shapiro–Wilk test. Gene expression was calculated using the ΔΔCt algorithm. Results have been presented as mean ± SD. The statistical significance was set at *p* < 0.05.

## 3. Results

### 3.1. The Chemical Composition of CM Extracts

The results of the chemical composition analysis for CM extracts are shown in Table 1. Five (iridoids, hydrolyzable tannins, anthocyanins, phenolic acid, and flavonols) and four (except for anthocyanin) phytochemical groups were identified in red and yellow extract samples, respectively. A detailed description of the identified compounds of CM was presented in the authors’ previous articles [18,19,23,24]. The extracts contained comparable amounts of iridoids, hydrolyzable tannins, phenolic acids, and flavonols, while the content of anthocyanins differed. The dominant compounds were iridoids (16,601.62 mg/100 g dw in the red extract and 16,922.32 mg/100 g dw in the yellow extract) and tannins (21,686.80 mg/100 g dw in the red extract and 18,722.01 mg/100 g dw in the yellow extract).

### 3.2. Viability Test

**Figure 1:**
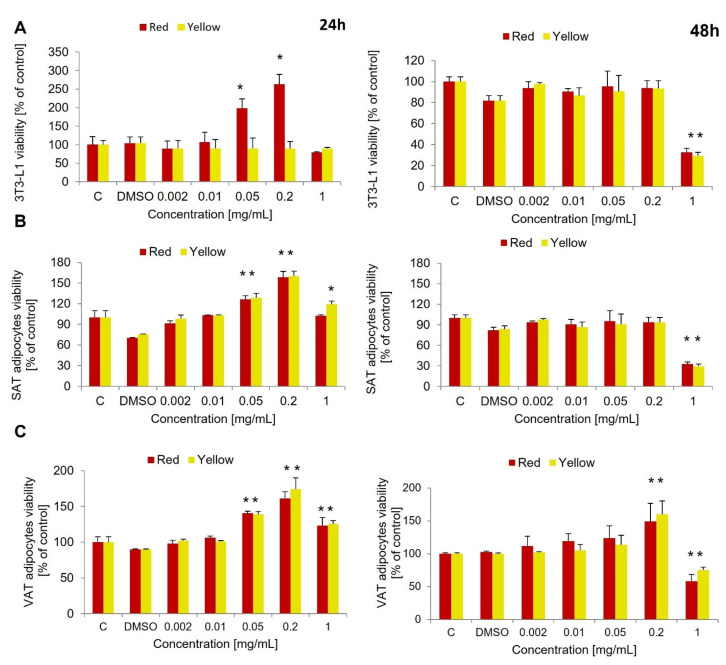
The viability test using 3-(4,5-dimethylthiazol-2-yl)-2,5-diphenyltetrazolium bromide (MTT) for red and yellow *Cornus mas* (CM) fruit extracts in various ranges of concentration performed after 24 h and 48 h in: (**A**) 3T3-L1 adipocytes, (**B**) SAT-derived human adipocytes (subcutaneous adipose tissue), (**C**) VAT-derived human adipocytes (visceral adipose tissue). DMSO-dimethyl sulfoxide. * *p* < 0.05, ** *p* < 0.000. Results have been presented as mean ± SD.

The viability test was conducted at two time points—at 24 h and 48 h—in all three groups of cells. In 3T3-L1 after 24 h, a significant increase in mitochondrial metabolism was revealed for cells treated with red CM fruit extract at a concentration of 0.05 mg/mL (*p* = 0.035) and 0.2 mg/mL (*p* = 0.002) compared to controls treated with DMSO. At the 48 h time point, a decreased cell viability was observed for cells treated with both red and yellow CM fruit extracts at a concentration of 1.0 mg/mL (both *p* < 0.000, Figure 1A).

In human SAT-derived adipocytes, the same effect of red CM fruit extract on cell viability was observed after 24 h, namely an increase in mitochondrial metabolism of MTT at a concentration of 0.05 mg/mL (*p* = 0.014) and 0.2 mg/mL (*p* = 0.001). A similar effect was observed for yellow CM fruit extract at a concentration of 0.05 mg/mL (*p* = 0.013), 0.2 mg/mL (*p* < 0.000) and 1.0 mg/mL (*p* = 0.035). On the other hand, after 48 h there was a decrease in SAT-derived adipocyte viability measured for the concentration of 1.0 mg/mL for both CM fruit extracts, red and yellow (*p* < 0.000 and *p* = 0.005, respectively, Figure 1B).

Similarly to SAT, VAT-derived adipocytes showed increased metabolic activity when treated with red and yellow CM fruit extracts for 24 h at the concentrations of 0.05 mg/mL (*p* = 0.001 for both extracts), 0.2 mg/mL (*p* < 0.000 and *p* = 0.001, respectively) and 1.0 mg/mL (*p* = 0.042 and *p* = 0.007, respectively). After 48 h, at the concentration of 0.2 mg/mL, there was still an increase in mitochondrial metabolism for both tested red and yellow CM fruit extracts (*p* = 0.048 and *p* = 0.006, respectively). However, a concentration of 1 mg/mL decreased the viability of the cells compared to controls with DMSO (*p* = 0.010 and *p* = 0.001, respectively, Figure 1C). Based on the viability test, the concentration of 0.01 mg/mL was selected for further study, as this concentration was observed to have no influence on cell viability or metabolism rate at the two time points tested. 

### 3.3. Glucose Uptake in Insulin-Resistant Adipocytes Treated with Red and Yellow CM Fruits Extracts

**Figure 2:**
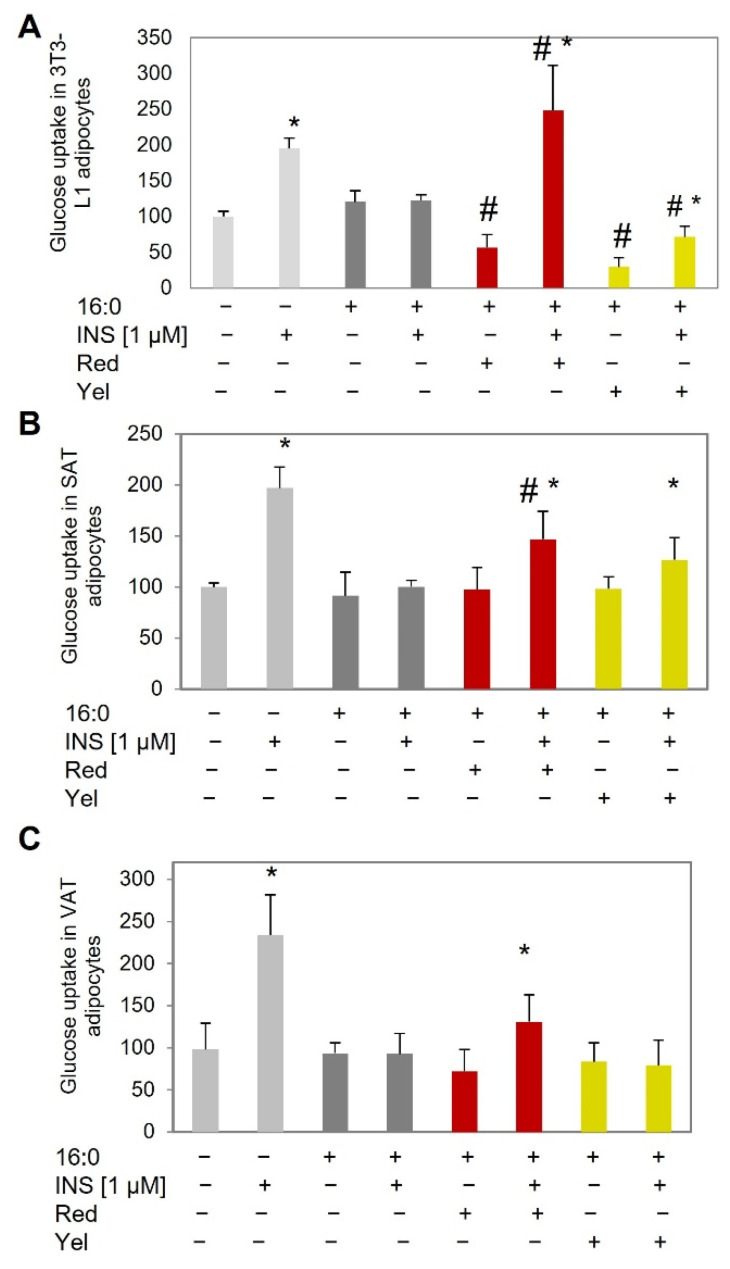
Glucose uptake test in control adipocytes and adipocytes with insulin resistance (induced using palmitic acid, 16:0) at baseline and after insulin stimulation (INS), and in IR adipocytes treated with red and yellow CM fruit extract at the concentration of 0.01 mg/mL in: (**A**) 3T3-L1 adipocytes, (**B**) SAT-derived human adipocytes, (**C**) VAT-derived human adipocytes. * *p* < 0.05 compared to the baseline (INS−) of the appropriate group of cells, ^#^ *p* < 0.05 compared to IR cells of the appropriate group (INS− or INS+). Results have been presented as mean ± SD.

Insulin resistance in adipocytes was induced with the use of palmitic acid (16:0) at a concentration of 0.5 mM. It has been proven that insulin resistance was successfully induced, and no increase in insulin-stimulated glucose uptake (ISGU) was observed in insulin-resistant adipocytes (Figure 2A–C). For all analyzed IR adipocytes ISGU was comparable to baseline glucose uptake and significantly lower than in control adipocytes (3T3L1 *p* = 0.002, SAT adipocytes *p* = 0.004, VAT adipocytes *p* < 0.000). In control cells, an increase in ISGU was observed compared to basal glucose uptake for all of the tested cells (3T3L1 *p* = 0.001, SAT adipocytes *p* = 0.003, VAT adipocytes *p* = 0.009) which proves the proper response of these cells to insulin. 

In 3T3-L1 insulin-resistant adipocytes, red CM fruit extract increased ISGU (*p* = 0.002, Figure 2B) compared to IR cells, which might suggest that red CM fruit extract might have reversed the previously developed insulin resistance. Similarly, yellow CM fruit extract increased ISGU (*p* = 0.02) compared to baseline glucose uptake; however, the increase was significantly lower than observed in IR cells (*p* = 0.005), which might suggest moderate activity against insulin resistance. Additionally, the baseline glucose uptake measured in adipocytes treated with red and yellow CM fruit extract was lower than in control and IR cells (*p* = 0.009 and *p* = 0.001, respectively) compared to IR adipocytes. 

In human SAT-derived adipocytes, both red and yellow CM fruit extracts increased ISGU in insulin-resistant adipocytes (*p* = 0.045 and *p* = 0.033, red and yellow, respectively, Figure 2B) compared to basal glucose uptake. However, the increase was slightly lower than the ISGU observed in control cells with proper insulin sensitivity. On the other hand, the observed ISGU was higher compared to ISGU in IR adipocytes, but only for red CM fruit extract was the increase statistically significant (*p* = 0.046, Figure 2B). For yellow CM fruit extract, ISGU was higher than in IR adipocytes, but not significantly so. 

In VAT-derived insulin-resistant adipocytes, a statistically significant increase in ISGU was observed only for red CM fruit extract (*p* = 0.016, Figure 2C) compared to basal glucose uptake. However, when compared to ISGU of IR cells, the increase was moderate, closely reaching significance (*p* = 0.053). Yellow CM fruit extract did not stimulate ISGU in VAT-derived insulin-resistant adipocytes and its level was significantly lower than observed for control cells (*p* = 0.001, Figure 2C), comparable to insulin-resistant cells. 

### 3.4. Insulin Signaling Gene Expression 

To investigate the possible mechanism by which red and yellow CM fruit extract could potentially drive insulin sensitivity in IR adipocytes, the expression of insulin pathway genes (*INSR*, *PIK3R1*, *SLC2A4*), insulin signaling gene (*PPARG*), inflammatory gene (*IL-6*), has been investigated. 

*INSR* gene expression was down-regulated in IR 3T3-L1 adipocytes and did not increase after incubation with CM fruit extracts (both red and yellow). The expression rate was similar to IR adipocytes at the basal stage and after insulin stimulation. In contrast to murine adipocytes, in SAT-derived adipocytes, an increase in *INSR* expression was observed in cells treated with red and yellow CM fruit extracts at the basal stage and after insulin stimulation compared to appropriate IR adipocytes (basal or insulin-stimulated). A statistically significant increase was observed for red CM fruit extract (*p* = 0.012 and 0.013 for the basal and insulin-stimulated stage, respectively) and yellow CM fruit extract (*p* = 0.005 and *p* = 0.002 for basal and insulin-stimulated glucose uptake) when compared to IR adipocytes. Similarly in IR VAT-derived adipocytes, the CM fruit extracts increased expression of the *INSR* gene by about two-fold (red CM fruit extract *p* = 0.012 and *p* = 0.011 for basal and insulin-stimulated stage, yellow CM fruit extract *p* = 0.005 for basal stage) comparing to IR adipocytes. 

The *SLC2A4* gene, encoding glucose transporter type 4 (GLUT4), was upregulated after insulin stimulation compared to non-stimulated cells for all of the investigated adipocytes (3T3-L1 *p* = 0.009, SAT adipocytes *p* = 0.024, VAT adipocytes *p* = 0.004). On the other hand, no changes in *SLC2A4* expression in IR adipocytes have been detected, which further proves the IR induced in these adipocytes. In IR 3T3-L1 adipocytes, an increase in *SLC2A4* expression was shown in cells treated with yellow CM extract (*p* = 0.016 and *p* = 0.038 for basal expression and after insulin stimulation) compared to IR adipocytes. 

An increase in expression of the *SLC2A4* gene in IR adipocytes cultured with CM fruit extracts has been observed also for SAT-derived adipocytes, both for red and yellow CM fruit extracts. Only in IR adipocytes treated with yellow CM extract the increase of *SLC2A4* expression was statistically significant in insulin-stimulated adipocytes compared to non-stimulated adipocytes (*p* = 0.050). Furthermore, in the IR SAT adipocytes treated with yellow CM fruit extract, the increase in *SLC2A4* gene expression after insulin stimulation was significantly higher compared to insulin-stimulated IR adipocytes (*p* = 0.001), and was similar to control insulin-sensitive adipocytes. For the red CM fruit extract, an increase in *SLC2A4* expression after insulin stimulation was lower than that observed for yellow CM fruit extract but was significantly higher than observed in IR adipocytes (*p* = 0.031). There were no changes in VAT-derived IR adipocytes, the expression of the *SLC2A4* gene was low compared to IR adipocytes treated with examined CM extracts and it did not increase after insulin stimulation. 

**Figure 3:**
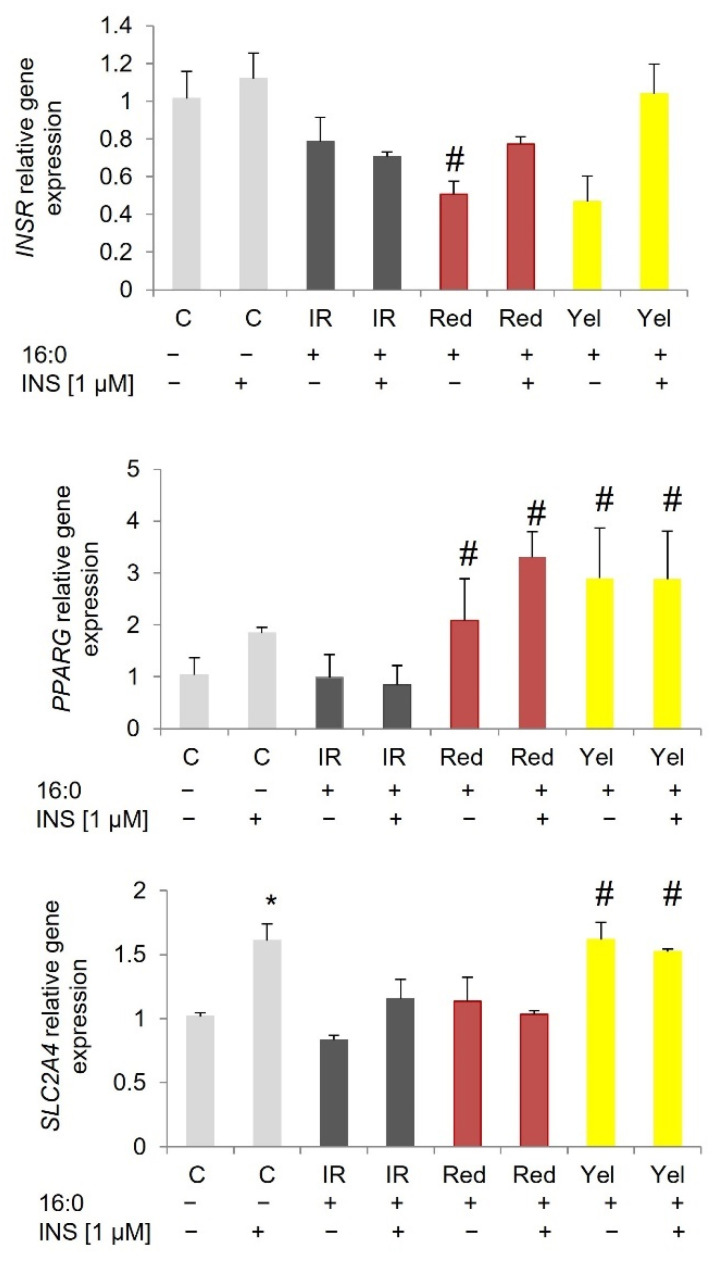
Gene expression in 3T3-L1 control adipocytes (C), insulin-resistant adipocytes (IR), and IR adipocytes treated with red and yellow (Yel) CM fruit extracts the concentration of 0.01 mg/mL. * *p* < 0.05 compared to the baseline (INS−) of the appropriate group of cells, ^#^ *p* < 0.05 compared to the corresponding IR adipocytes (baseline INS− and after insulin stimulation INS+). Results have been presented as mean ± SD.

*PPARG* was overexpressed in control 3T3-L1 adipocytes after insulin stimulation (not statistically significant), SAT adipocytes (*p* = 0.024), and VAT adipocytes (*p* = 0.004) compared to baseline expression. In all cells treated with CM fruit extracts, the expression of *PPARG* increased both at the basal stage and after insulin stimulation. In 3T3-L1 adipocytes, *PPARG* was three times higher in adipocytes treated with red CM fruit extract at baseline (*p* = 0.001) and after insulin stimulation (*p* < 0.000) compared to appropriate IR adipocytes. Similarly, *PPARG* was overexpressed in IR 3T3-L1 adipocytes treated with yellow CM fruit extract at baseline (*p* = 0.009) and after insulin stimulation, (*p* = 0.001) compared to the corresponding IR adipocytes (Figure 3). 

**Figure 4:**
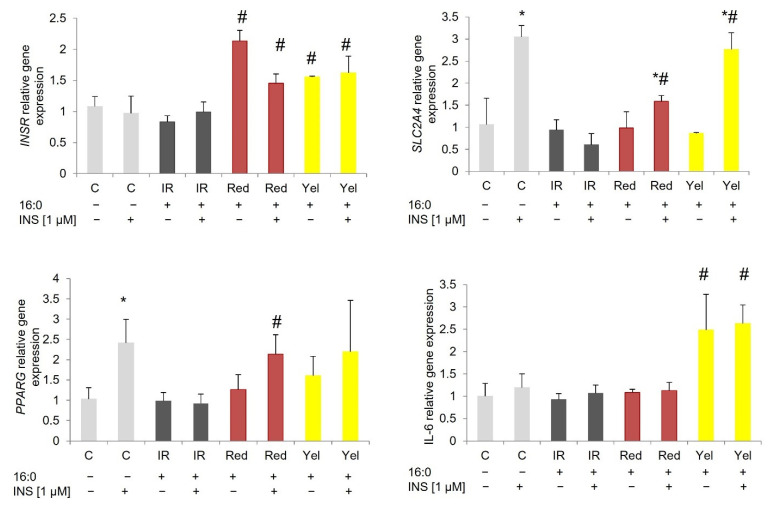
Gene expression in SAT-derived human control adipocytes (C), insulin-resistant adipocytes (IR), and IR adipocytes treated with Red and Yellow (Yel) CM fruit extracts the concentration of 0.01 mg/mL. * *p* < 0.05 compared to the baseline (INS−) of the appropriate group of cells, ^#^ *p* < 0.05 compared to the corresponding IR adipocytes (baseline INS− and after insulin stimulation INS+). Results have been presented as mean ± SD.

In SAT-derived insulin-resistant adipocytes, the expression of *PPARG* was higher in insulin-stimulated cells treated with red and yellow CM fruit extracts as compared to insulin-stimulated IR cells; however, only for red CM fruit extract was the increase statistically significant (*p* = 0.037). The basal expression of *PPARG* was slightly increased in insulin-resistant adipocytes treated with red and yellow CM fruit extracts, but without statistical significance (compared to basal expression of IR adipocytes). On the other hand, no increase in *PPARG* expression was observed for IR SAT adipocytes; the expression after insulin stimulation was low compared to the baseline and significantly lower compared to insulin-stimulated control adipocytes (*p* = 0.003, Figure 4). 

**Figure 5:**
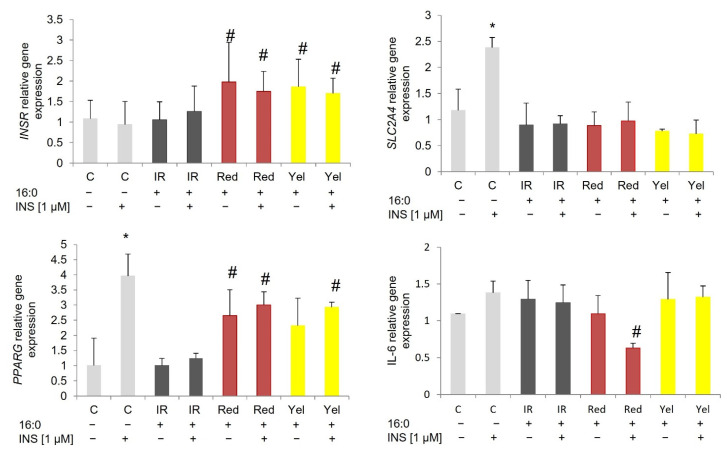
Gene expression in VAT-derived human control adipocytes (C), insulin-resistant adipocytes (IR), and IR adipocytes treated with Red and Yellow (Yel) CM the concentration of 0.01 mg/mL. * *p* < 0.05 compared to the baseline (INS−) of the appropriate group of cells, ^#^ *p* < 0.05 compared to the corresponding control cells (baseline INS- and after insulin stimulation INS+). Results have been presented as mean ± SD.

In VAT-derived IR adipocytes after insulin stimulation, the level of *PPARG* expression was low, similar to the baseline, and significantly lower (*p* = 0.005, Figure 5) compared to insulin-stimulated control adipocytes). On the other hand, the expression of *PPARG* increased about 2–3 times in IR adipocytes treated with CM fruit extracts both at baseline and after insulin stimulation. The red CM fruit extract increased the baseline expression of *PPARG* for red CM extracts (*p* = 0.037) and after insulin stimulation (*p* = 0.045); for yellow CM fruit extract, the increase was observed only in insulin-stimulated adipocytes (*p* = 0.014) when compared to corresponding IR adipocytes.

Additionally, overexpression of *IL-6* has been observed in SAT-derived insulin-resistant adipocytes treated with yellow CM fruit extract. The increase was observed both at baseline (*p* = 0.026) and after insulin stimulation (*p* = 0.005, Figure 4) compared to the corresponding IR adipocytes. No changes in *IL-6* expression were observed for other cells.

## 4. Discussion

In the present paper, we have shown that the addition of CM fruit extracts overcomes insulin resistance and increases glucose uptake after insulin stimulation in all experimental cell lines; however, a more favorable effect has been observed for red CM fruit extract. The glucose uptake stimulated by yellow CM fruit extract was not significant, although a trend of increasing uptake was evident. In cells supplemented with red CM fruit extract, after stimulation with insulin, glucose absorption increases at least four-fold, and in cells supplemented with yellow CM fruit extract, after stimulation with insulin, at least a two-fold increase was observed, comparing to basal glucose uptake, depending on the cell types. Furthermore, we have displayed increased expression of *PPARG*, the main transcription factor, regulating adipogenesis and phenotype of mature adipocytes in all types of cells treated with red and yellow CM fruit extract. 

As a significant increase in glucose uptake has been demonstrated in experimental cells, the next step was to identify a potential mechanism that might be responsible for overcoming insulin resistance previously developed in adipocytes. For this purpose, the measurement of the expression of genes involved in insulin signal transmission was investigated. The study showed the effect of CM fruit extracts on the expression of the aforementioned genes that increased in adipocytes stimulated with insulin in control cells and cells treated with CM fruit extracts. Based on the obtained results, it can be concluded that CM fruits might affect the expression of the investigated genes of the insulin pathway which might be connected with improving insulin action and breaking down insulin resistance artificially induced in these cells by palmitic acid. 

Numerous data have been reported demonstrating the use of CM extracts from various parts of the plant as antiglycemic, antihyperlipidemic agents with antidiabetic and antiobesity properties. Most of the studies used rats, rabbits, or hamsters as the study model. The decrease in blood glucose level, TG content, accumulation of lipids in the liver, and body weight was reported in rats treated with CM extracts [25,26,27]. Furthermore, the observed effect of CM fruit extract was similar to the regularly prescribed antidiabetic drug glibenclamide. Similar antiglycemic and antidiabetic effects were observed for other animals used in studies, i.e., mice [28], rabbits [12,17], and hamsters [29]. Furthermore, a decreased blood glucose level correlated with an increased rate of insulin secretion. That in vitro observation is similar to the results of the presented study, where a higher glucose uptake by adipocytes has been observed in cells treated with CM fruit extracts of different red- and yellow-fruit cultivars. Furthermore, the same effect was observed for all tested experimental cells, both murine and human. Furthermore, CM fruit extracts not only increased glucose uptake rate in the tested cells but were also able to reverse previously developed insulin resistance and restore proper insulin signaling. 

Some of the previously published reports attempted to indicate the mechanism of action of CM fruit extract. It is well known that CM fruits are a rich source of biologically active compounds [6,9], with possible broad action within a single cell and the whole organism [30,31]. It is important to understand the mechanism of action of each individual compound of CM fruits, as well as the effect of the combination of compounds found in the whole fruit. 

Insulin sensitivity is regulated via different mechanisms including hormonal regulation. It is estimated that in patients with hormonal abnormalities, for example, hypothyreosis or polycystic ovary syndrome (PCOS), the proper insulin response is abolished. However, no changes in hormone levels have been associated with CM fruit extracts [25]. It is more likely that the mechanism might involve gene regulation and signal transduction. Indeed, the present paper and other studies showed that CM fruit extract increases the expression rate of numerous transcription factors involved in lipid metabolism as well as insulin sensitivity regulation, including *PPARA*, *PPARG* and *LXR* [12,13,32]. 

The nuclear peroxisome proliferator-activated receptor gamma (*PPARG*) is a key transcription factor regulating the development and proper metabolism of adipocytes. Additionally, *PPARG* may influence the regulation of other genes important for adipogenesis and the insulin pathway [33,34]. Moreover, *PPARG* regulates the expression of adiponectin—responsible for increasing the sensitivity of cells to insulin [33,34]. Earlier studies on rabbits have shown that compounds isolated from CM fruit extract increase the expression of *PPARA* and *PPARG* receptors [12] and indicate an important role of *PPARG* in the regulation of potential metabolic disorders. The presented study also confirmed a significant increase in *PPARG* expression in cells supplemented with CM fruits. The mechanism of reversing insulin resistance in the investigated cells likely results from the stimulation of *PPARG* expression after the addition of CM fruits. Numerous studies confirm the reduction of *PPARG* expression in insulin resistance both in studies on cell lines and in human studies [21,35]. In addition, *PPARG* agonists represent an option for the treatment of insulin resistance in type 2 diabetes. Furthermore, the present authors have recently revealed that *PPARG* is the first gene abnormally expressed as a result of induction of insulin resistance, which proves its crucial role in the regulation of insulin sensitivity [20].

It is worth mentioning that a similar effect, increased expression of *PPARG,* was observed in numerous tissues and organs, including the liver [32], aortic endothelial cells [13], and adipocytes. Those results indicate the universality of the health-promoting effect of CM fruits on the entire body through the wide-range regulation of metabolism, from lipid metabolism to the hypoglycemic effect.

However, subtle differences in effect have been observed depending on the type of CM fruit both for the stimulation of glucose uptake and the expression of key genes of the insulin pathway. First, red CM fruit extract increased insulin-stimulated glucose uptake at a higher rate than the yellow extract, which might be due to the content of biologically active compounds, mainly anthocyanins. It has been shown previously that anthocyanins highly increased the expression of *PPARG* in the liver [32]. Although the present study did not detect substantial differences in the expression of *PPARG* in the examined adipocytes cultured with red and yellow CM fruit extracts, insulin-stimulated glucose uptake was higher in cells treated with anthocyanin-rich CM extract. It may suggest that anthocyanins could facilitate glucose uptake via various pathways. Furthermore, the responses to CM fruit extract were slightly different in SAT and VAT adipocytes, and insulin-resistant SAT adipocytes showed a better response in terms of insulin signaling and glucose uptake than insulin-resistant VAT adipocytes. The above-mentioned differences were observed in previous research, which proves that SAT and VAT adipocytes differ in terms of metabolism and pathogenesis of metabolic disorders [21]. 

The effect of CM fruit extracts on mitochondrial metabolism also needs pointing out. It was demonstrated that mitochondrial metabolism rate increased for both CM fruits extracts after 24 h. Thus, further analysis needs to be performed to evaluate the effect of CM fruit extract on mitochondrial metabolism, and if the effect might result from improving oxidative phosphorylation other mechanisms, for example, the elimination of reactive oxygen species or mitochondrial biogenesis in the treated cells. Further research needs to be performed to evaluate whether CM fruit extract might improve the overall cell metabolism, including insulin signaling, by influencing mitochondria. 

The present study had some limitations, including a low number of human SAT and VAT-derived adipocytes, and the fact that all MSC donors were men. The other limitation might be insulin resistance induction using palmitic acids. The real pathomechanism of insulin resistance is more complex, involving lipid dysregulation, hyperinsulinemia, and an inflammatory state. All of these disorders lead to dysregulation in carbohydrate metabolism and insulin resistance induction. In the present study, insulin resistance induction did not correspond to the observed in vivo mechanism of the pathogenesis of IR. For that reason, the mechanism of restoring proper insulin sensitivity in insulin-resistant adipocytes in vivo must be evaluated in an in vivo model to evaluate the benefits to the whole organism.

To conclude, studies have shown that CM fruits extract can reverse insulin resistance and have a positive effect on cell metabolism. Moreover, the potential mechanism of the influence of these fruits may be related, as in the case of thiazolidinediones, to the upregulation of nuclear gamma proliferator-activated receptor (*PPARG*). Extending the research to include the expression of other genes involved in the transmission of the insulin signal or regulating insulin sensitivity in adipocytes will allow a more accurate understanding of the mechanisms of reversing the existing insulin resistance by CM compounds. Further research using human and animal cells will help elucidate the relationship between CM fruit consumption and type 2 diabetes prevention, which could lead to the development of effective dietary supplements and foods in the future.

## Figures and Tables

**Table 1:** Characteristic (mg/100 g dry weight (dw)) of purified extracts from red (red extract) and yellow (yellow extract) cultivars of CM fruits.

Phytochemical Groups	Red Extract	Yellow Extract
Iridoids	16,601.62 *	16,922.32
Hydrolysable tannins	21,686.80	18,722.01
Anthocyanins	2201.49	0.00
Phenolic acids	697.73	1055.56
Flavonols	240.83	196.48

* Total of compounds.

## Data Availability

Not applicable.

## Supplementary Materials

The following supporting information can be downloaded at: https://www.mdpi.com/article/10.3390/nu14112307/s1, Figure S1: The undifferentiated and fully mature adipocytes: 3T3-L1, SAT-derived adipocytes, and VAT-derived adipocytes; Figure S2: The expression of genes encoding transcription factors regulating adipogenesis: *CEBPA* and *PPARG* in preadipocytes (D0) and fully mature adipocytes (D10).
