# Supplementary material for: Cornus mas L. Increases Glucose Uptake and the Expression of PPARG in Insulin-Resistant Adipocytes
Source: Nutrients. 2022 May 31;14(11):2307. doi: 10.3390/nu14112307 (PMC9183168; doi:10.3390/nu14112307)
Supplement: Supplementary file 1 [file nutrients-14-02307-s001.zip › nutrients-1721984-supplementary.pdf]

Supplementary Materials

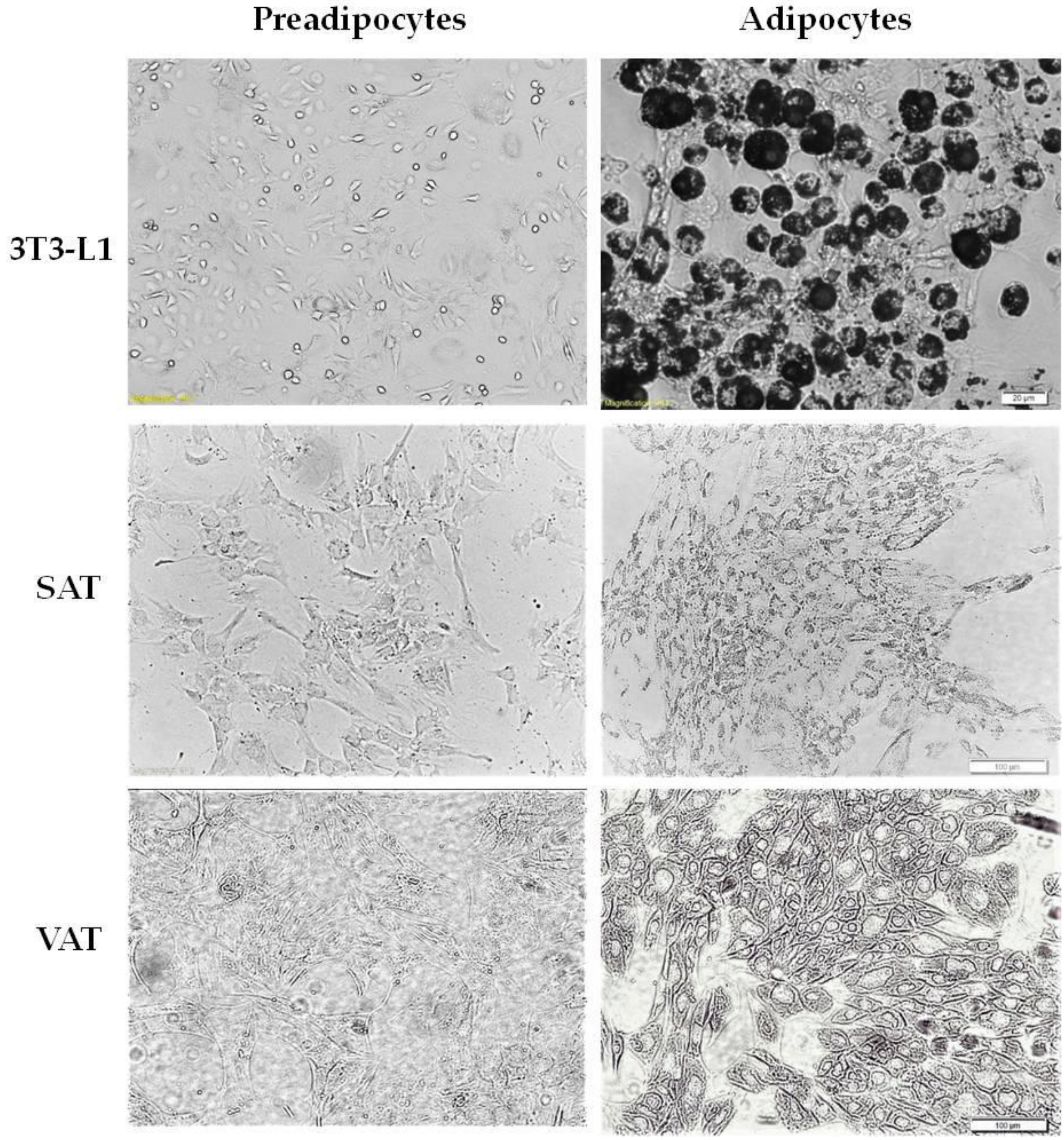

Figure S1. The undifferentiated and fully mature adipocytes: 3T3-L1, SAT-derived adipocytes, and VAT-derived adipocytes.

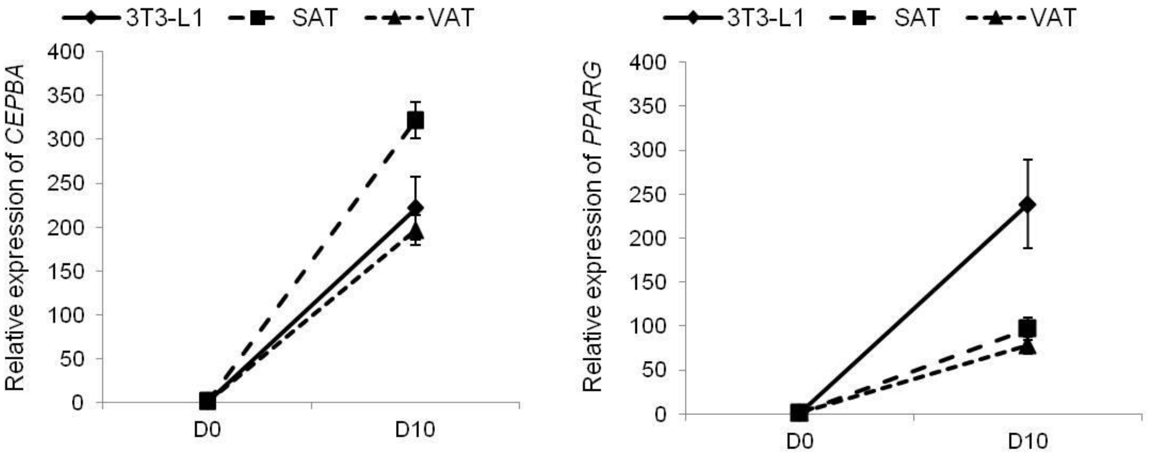

Figure S2. The expression of genes encoding transcription factors regulating adipogenesis: *CEBPA* and *PPARG* in preadipocytes (D0) and fully mature adipocytes (D10).
